# Genetic Analysis of a Novel H16N3 Virus Isolated from a Migratory Gull in China in 2021 and Animal Studies of Infection

**DOI:** 10.1128/spectrum.02484-22

**Published:** 2022-10-31

**Authors:** Yanwen Wang, Hong Zhang, Mengjing Wang, Jing Guo, Conghui Zhao, Pengfei Cui, Guohua Deng, Dong Chu, Jinyan Shen, Xiaohong Sun, Xinxin Gao, Yubao Li, Wenqiang Liu, Peng Peng, Xuyong Li

**Affiliations:** a College of Agronomy, Liaocheng University, Liaocheng, China; b Harbin Veterinary Research Institute of Chinese Academy of Agricultural Sciences, State Key Laboratory of Veterinary Biotechnology, National Poultry Laboratory Animal Resource Center, Harbin, China; c Biological Disaster Control and Prevention Center, National Forestry and Grassland Administration, Shenyang, China; University of Georgia

**Keywords:** avian influenza viruses, H16N3, mice, poultry, wild birds

## Abstract

H16 avian influenza viruses mainly circulate in wild migratory gulls worldwide, and the infection risks in poultry and mammals remain largely unknown. In this study, we isolated a novel H16N3 virus from migratory gulls in eastern China in 2021. Genetic analysis indicated that the H16N3 virus originated from the H16 and H13 viruses that circulated in wild birds. This H16N3 virus has not adapted to replicate in chickens, ducks, or mice, although it can be transmitted between inoculated and contacted birds. The circulation of H16Nx viruses in the Northern Hemisphere indicates that we should strengthen active surveillance to monitor their prevalence and evolution in migratory gulls and their introduction into other migratory and domestic waterfowl.

**IMPORTANCE** Migratory wild birds are natural reservoirs of H16 viruses and play a key role in the global prevalence of these viruses. Here, we found that H16 viruses predominantly circulate in migratory gulls and that the gull H16N3 virus cannot replicate efficiently in chickens, ducks, or mice without prior adaptation. These findings contribute to our understanding of the ecology, evolution, and biological properties of H16 viruses and will guide avian influenza surveillance in birds.

## OBSERVATION

Avian influenza viruses are classified into 16 hemagglutinin (HA) and 9 neuraminidase (NA) subtypes according to differences in antigenicity and genetic profiles. To date, several important subtypes, mainly H5N1, H5N6, H5N8, H7N7, H7N9, H9N2, H10N3, and H10N8, which originated in wild birds or poultry have caused substantial economic losses to the poultry industry and pose an increasing threat to human health (1–8). Identification and characterization of novel subtypes of avian influenza viruses that emerge in migratory birds will guide early warnings about the risks of cross-species infection and transmission of such viruses. H16 viruses are predominantly prevalent in migratory birds, especially gulls, and are rarely isolated from domestic poultry. Consequently, the genetic profiles and replication and transmission characteristics of the H16 viruses circulating in the past 2 decades in domestic birds and mammals are largely unknown (9–14). In this study, we isolated a novel H16N3 virus from fecal droppings of migratory gulls in eastern China in 2021. We performed a detailed evolutionary analysis and assessed replication and transmission of the novel H16N3 virus in domestic chickens and ducks as well as virulence in mice. Additionally, the global spatiotemporal prevalence of the H16 viruses in their natural reservoir hosts was statistically analyzed and mapped to fully understand the ecology of H16 viruses.

**FIG 1:**
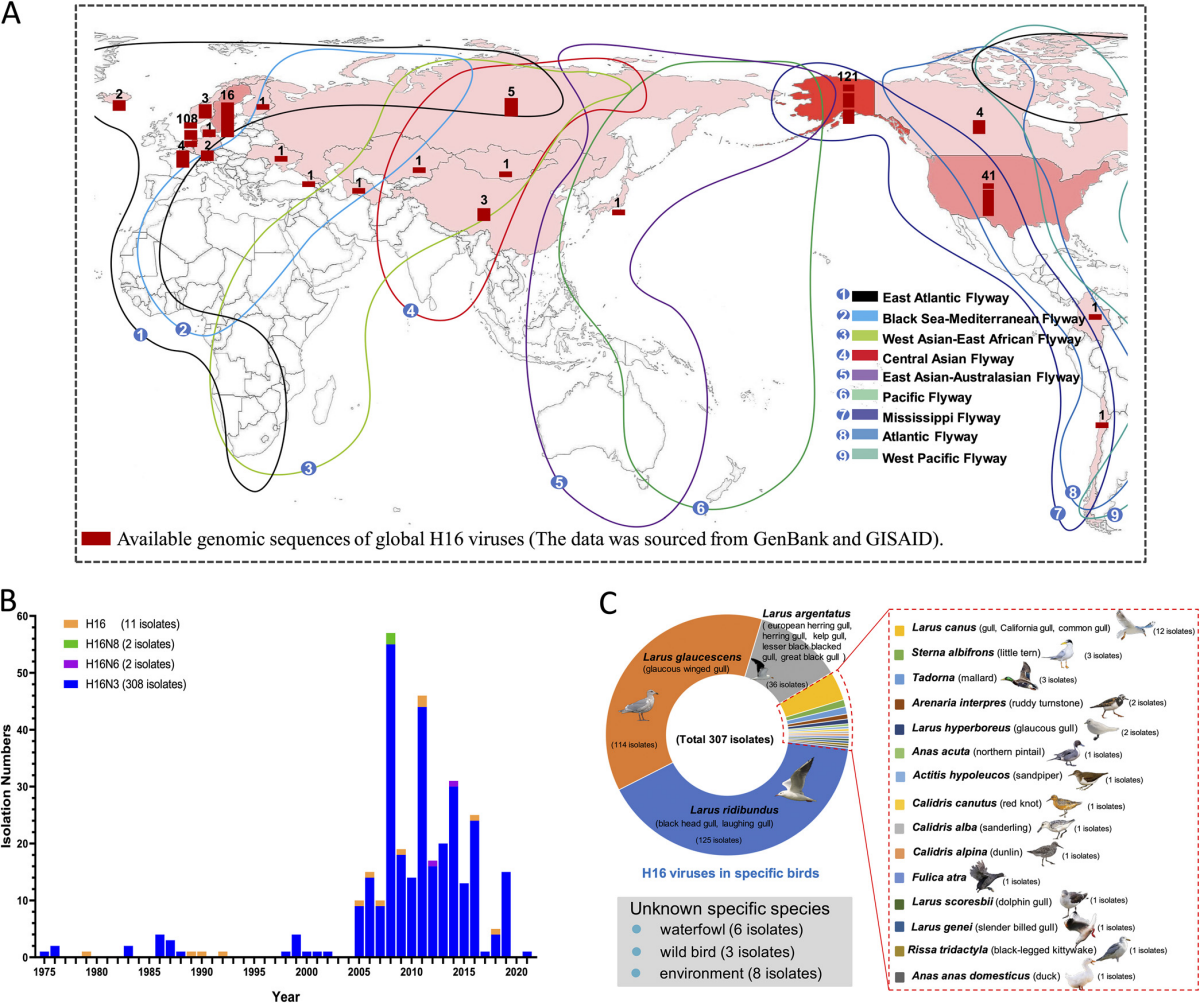
Global circulation, annual numbers of isolates, and prevalence of H16 viruses in wild and domestic birds. (A) Global circulation of H16 viruses. The HA sequences were downloaded from the GenBank and GISAID databases, and then the isolation locations were statistically analyzed and noted on the map. (B) Number of H16 viruses isolated annually. (C) H16 viruses in different birds. The numbers of H16 virus isolates in different birds are shown according to their submission information in GenBank and GISAID. The map is sourced from Standard Map Service (http://bzdt.ch.mnr.gov.cn/index.html).

Understanding the global prevalence of H16 viruses in different species will help the development of targeted epidemiological surveillance. In this study, we downloaded all the sequences of the HA and NA genes of H16Nx viruses from the GenBank and GISAID databases. According to the geographical distribution of the H16 isolates, H16 viruses have been mainly detected in the Northern Hemisphere, including North America, northern Europe, and East Asia. A total of 162 HA sequences and 108 HA sequences of H16 viruses from North America and the Netherlands, respectively, have been submitted to the databases. Only three H16 viruses isolated from gulls, including two H16N3 viruses, were isolated from great black-headed gulls in western China in 2018 by Li et al., and the H16N3 virus isolated in this study has been reported in China (Fig. 1A) (9). We categorized the subtypes of the H16Nx isolates from 1975 to 2021 and found that the H16 viruses with available data could be classified into H16N3, H16N6, and H16N8 subtypes. Notably, H16N3 was the predominant subtype, for which 308 isolates were identified (Fig. 1B). We further enumerated and classified the hosts of the H16 viruses and found that gulls are primary natural reservoirs of H16 viruses. A total of 291 of the 307 isolates were detected in different gull species, including Larus ridibundus (125 isolates), Larus glaucescens (114 isolates), Larus argentatus (36 isolates), Larus canus (12 isolates), and others (4 isolates). Sixteen of the 307 isolates were detected in other migratory birds, including mallards, ruddy turnstones, northern pintails, dunlins, and ducks (Fig. 1C). These statistical data indicate that H16N3 is the predominant H16Nx subtype in the Northern Hemisphere and occurs mainly in migratory gulls.

From October 2017 to February 2022, we conducted annual avian influenza virus surveillance in wetland of migratory wild bird habitats along the East-Australasian migratory flyway in eastern China. The samples were identified by PCR with specific M and HA (H5, H7) primers. The suspected H5- or H7-positive samples were transferred to an enhanced animal biosafety level 3 (ABSL-3) facility in the Harbin Veterinary Research Institute of the Chinese Academy of Agricultural Sciences for further virus identification and isolation, while the remaining suspected positive samples were injected into 10-day-old embryonated chicken eggs to isolate the viruses in an ABSL-2 laboratory at Liaocheng University. One H16N3, four H3N8, one H6N1, and six H13N6 viruses were isolated from 312 fecal dropping samples from migratory gulls in the Yellow River delta wetland on 10 October 2021. We named the virus A/gull/W1359/Shandong/2021(H16N3), sequenced the open reading frame (ORF) of the virus by Sanger sequencing, and characterized its molecular markers (GISAID accession numbers EPI2181372 to EPI2181379). Amino acid sequences of SINER/GLF were observed at the cleavage site in HA, which is typical of viruses with low pathogenicity in poultry. Several amino acid substitutions that contribute to increased virulence or transmissibility of avian influenza viruses were detected in the H16N3 virus, including PB1 207K, PB1 436Y, PA 515T, M1 30D, and M1 215A (15, 16). Subsequently, Bayesian time-resolved phylogenetic trees for the ORFs of the HA and NA genes of the H16N3 viruses (including the related sequences downloaded from GISAID and GenBank) were constructed to elucidate their genesis and evolutionary timeline. We found that the HA genes of the H16N3 viruses formed several branches, and the HA gene of the novel virus detected in the current study clustered on the same branch as HA of H16N3 viruses circulating in Eurasia; moreover, it shared 95.6% nucleotide identity with the isolates with the highest homology (see Fig. S1A and Table S1 in the supplemental material). The NA genes of the H16N3 viruses formed at least seven branches on the phylogenetic tree. The NA gene of the novel H16N3 virus detected in this study was clustered on a separate branch with those of viruses detected in Japan and the Netherlands and had less than 95.9% nucleotide similarity with the viruses on the same branch (Fig. S1B and Table S1). Phylogenetic analysis indicated that the internal genes PB2, PB1, PA, NP, and NS of H16N3 virus originated from H13N6 and H13N8 viruses that were mainly isolated from different gulls in Eurasia, North America, and Alaska. The M gene of the H16N3 virus clustered on a branch with those of H16N3 viruses that were detected in gulls in the Netherlands in 2013 and 2017 and shared 98.9% homology with the most closely related viruses (Fig. S2 and Table S1).

**FIG 2:**
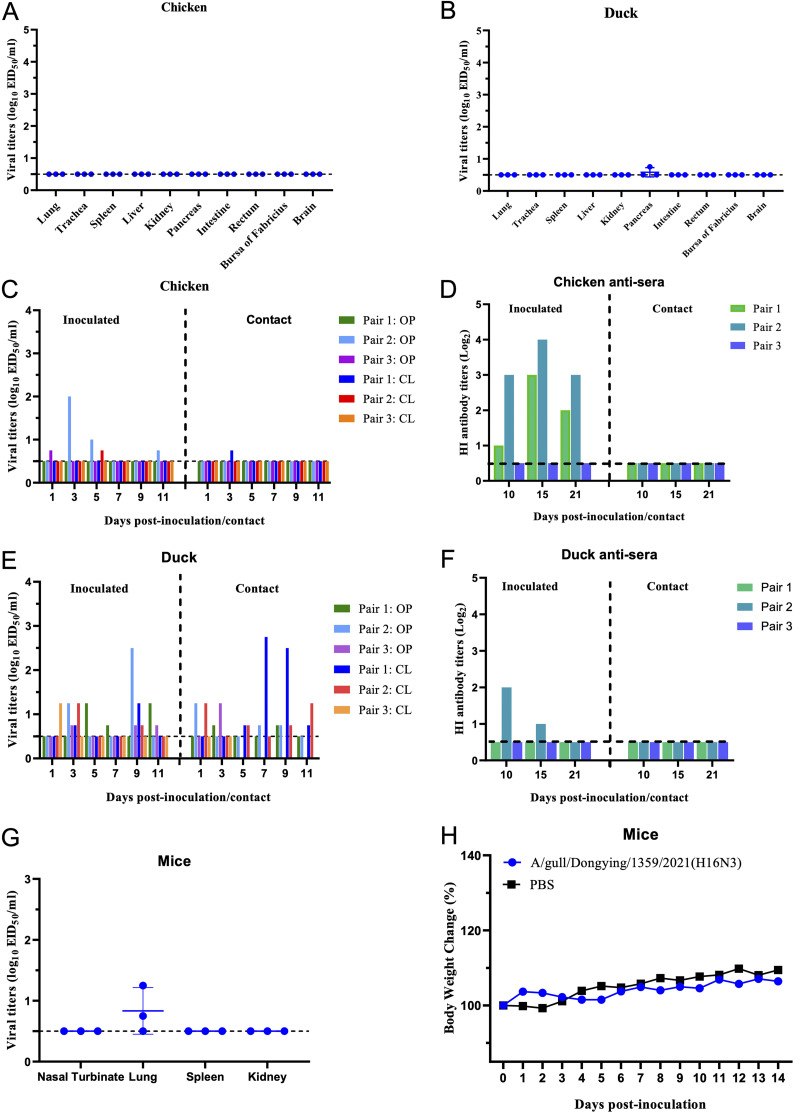
Replication and transmission of the H16N3 virus in chickens, ducks, and mice. (A and B) Replication of the H16N3 virus in chickens and ducks. Three chickens and ducks were inoculated with the virus, and titers in organ tissues were measured in eggs at 3 days p.i. The dashed line indicates the lower detection limit. (C and E) Transmission of the H16N3 virus in chickens and ducks. Three chickens and ducks were inoculated with the virus, and another naive bird was placed into the same cage as the contact group at 24 hpi. Oropharyngeal and cloacal swabs were collected from each bird, and titers were measured in eggs. The horizontal dashed line indicates the lower detection limit. (D and F) HI antibody titers in chicken and duck antisera. Serum samples from chickens and ducks in the inoculated and contact groups were collected at 10, 15, and 21 days p.i., and HI antibody titers were tested with the HI assay. (G) Replication of the H16N3 virus in mice. The mice were inoculated with the virus, and titers in organ samples were measured in eggs at 3 days p.i. The dashed line indicates the lower detection limit. (H) Body weight changes in mice. The mice were inoculated with the virus and then observed for 2 weeks to monitor clinical symptoms and body weight changes. PBS, phosphate-buffered saline.

The introduction of avian influenza viruses of wild bird origin into domestic waterfowl and poultry populations will increase epidemic risks due to replication and transmissibility alterations (11, 17–19). However, the infectivity and replication properties of the currently circulating H16N3 viruses have not been fully investigated. In a previous study, the gull-derived H16N3 virus was found to cause limited infection in chickens (20). Fereidouni et al. found that mallard ducks were resistant to infection with H16N3 viruses (10). Here, we evaluated replication of the GL/1359/21 virus in specific-pathogen-free (SPF) chickens and ducks. We first evaluated the replication of the gull virus in embryonated chicken eggs before the animal studies and found that the virus replicated in chicken eggs, with viral HA titers (log_2_) of 6 to 7 at 24 or 48 h postinfection (hpi) (Fig. S3). The chickens and ducks were inoculated intranasally with the virus at 10^6^ 50% egg infective doses (EID_50_) in a volume of 100 μL and were euthanized on day 3 postinfection (p.i.), and tissues from lung, trachea, spleen, liver, kidney, pancreas, intestine, rectum, bursa of Fabricius, and brain were collected to determine viral titers. No virus was detected in any organs or tissues of the infected chickens (Fig. 2A), and the virus was detected at a low titer in the pancreas of only one infected duck (Fig. 2B). We then evaluated the transmission ability of the H16N3 virus in both chickens and ducks by calculating titers in oropharyngeal and cloacal swabs from inoculated and contacted birds. Interestingly, low titers of virus were detected in two inoculated chickens and one contacted chicken, and the virus was detected in all three inoculated and contacted ducks (Fig. 2C and E). Hemagglutination inhibition (HI) assays indicated that the sera of two inoculated chickens were positive for the H16N3 virus, with titers (log_2_) ranging from 1 to 4 at 10, 15, and 21 days p.i. (Fig. 2D); however, only one inoculated duck was positive for the H16N3 virus, with HI antibody titers (log_2_) of 2 and 1 at 10 and 15 days p.i., respectively (Fig. 2F). These experimental studies indicate that the H16N3 virus of wild bird origin is poorly adapted to replicate in chickens and ducks, although the H16N3 virus can be transmitted efficiently among ducks.

To further understand the potential threat of the H16N3 virus to mammals, we evaluated replication of the virus in mice. Eight SPF BALB/c mice were inoculated with the virus at 10^6^ EID_50_ in a volume of 50 μL, and three mice were euthanized to analyze viral titers in the nasal turbinate, lungs, spleen, and kidneys. Body weight changes and clinical symptoms in the other five mice were observed for 14 days p.i. The virus was detected in the lung tissues of two inoculated mice, with low titers at 3 days p.i. (Fig. 2G). The mice inoculated with the H16N3 virus did not lose any body weight and did not show any clinical symptoms during the 2-week observation period (Fig. 2H). In a previous study, Li et al. isolated two H16N3 viruses from the great black-headed gull and found that these viruses could not replicate in mice and preferentially bound to avian-type receptors (9). These results indicate that the naturally isolated H16N3 virus in gulls needs to undergo further adaptation before it can replicate successfully in mice.

In summary, we isolated a novel H16N3 virus from migratory gulls in eastern China and characterized its molecular and evolutionary properties. Animal studies of natural reservoirs and statistical analysis indicated that as the predominant subtype of H16Nx viruses, H16N3 viruses primarily circulate in migratory gulls and have not adapted to replicate efficiently in chickens, ducks, or mice. In the future, viral surveillance among gulls and other migratory waterfowl should be strengthened to monitor the circulation and evolution of H16Nx at the wild and domestic waterfowl interface.

### Data availability.

The whole-genome sequences of the H16N3 virus reported in this study have been deposited into GISAID (https://www.gisaid.org/). The accession numbers of eight gene sequences are EPI2181372 to EPI2181379.
