# Supplementary material for: Genetic Analysis of a Novel H16N3 Virus Isolated from a Migratory Gull in China in 2021 and Animal Studies of Infection
Source: Microbiol Spectr. 2022 Oct 31;10(6):e02484-22. doi: 10.1128/spectrum.02484-22 (PMC9769943; doi:10.1128/spectrum.02484-22)
Supplement: Supplemental file 1 — Table S1, Fig. S1 to S3, and supplemental materials and methods. Download spectrum.02484-22-s0001.pdf, PDF file, 1.9 MB [file spectrum.02484-22-s0001.pdf]

Supplementary information

Table S1. Genetic similarity of A/gull/Shandong/1359/2021 (H16N3) with the most related avian influenza viruses in the database.

| Gene segments | The most related isolates in Genbank and GISAID | Nucleotide identity | Accession numbers |
|---|---|---|---|
| HA | A/black-headed gull/Netherlands/2/2015(H16N3) | 95.6% | KX978663.1[a] |
| NA | A/duck/Hokkaido/WZ82/2013(H16N3) | 95.9% | LC339709.1[a] |
| PB2 | A/Chroicocephalus ridibundus/Belgium/13464/2020(H13N8) | 97.4% | EPI1942887[b] |
| PB1 | A/black-headed gull/Netherlands/5/2014(H13N6) | 97.8% | KX979636.1[a] |
| PA | A/shelduck/Ukraine/KT-9-2-11/2016(H13N6) | 98.3% | MW132948.1[a] |
| NP | A/shelduck/Ukraine/KT-9-2-11/2016(H13N6) | 96.2% | MW132951.1[a] |
| M | A/black-headed Gull/Netherlands/5/2017(H16N3) | 98.9% | MK192343.1[a] |
| NS | A/shelduck/Ukraine/KT-9-2-11/2016(H13N6) | 98.0% | MW132950.1[a] |

[a] The accession numbers of Genbank were listed.

[b] The accession number of GISAID was listed.

Fig.S1

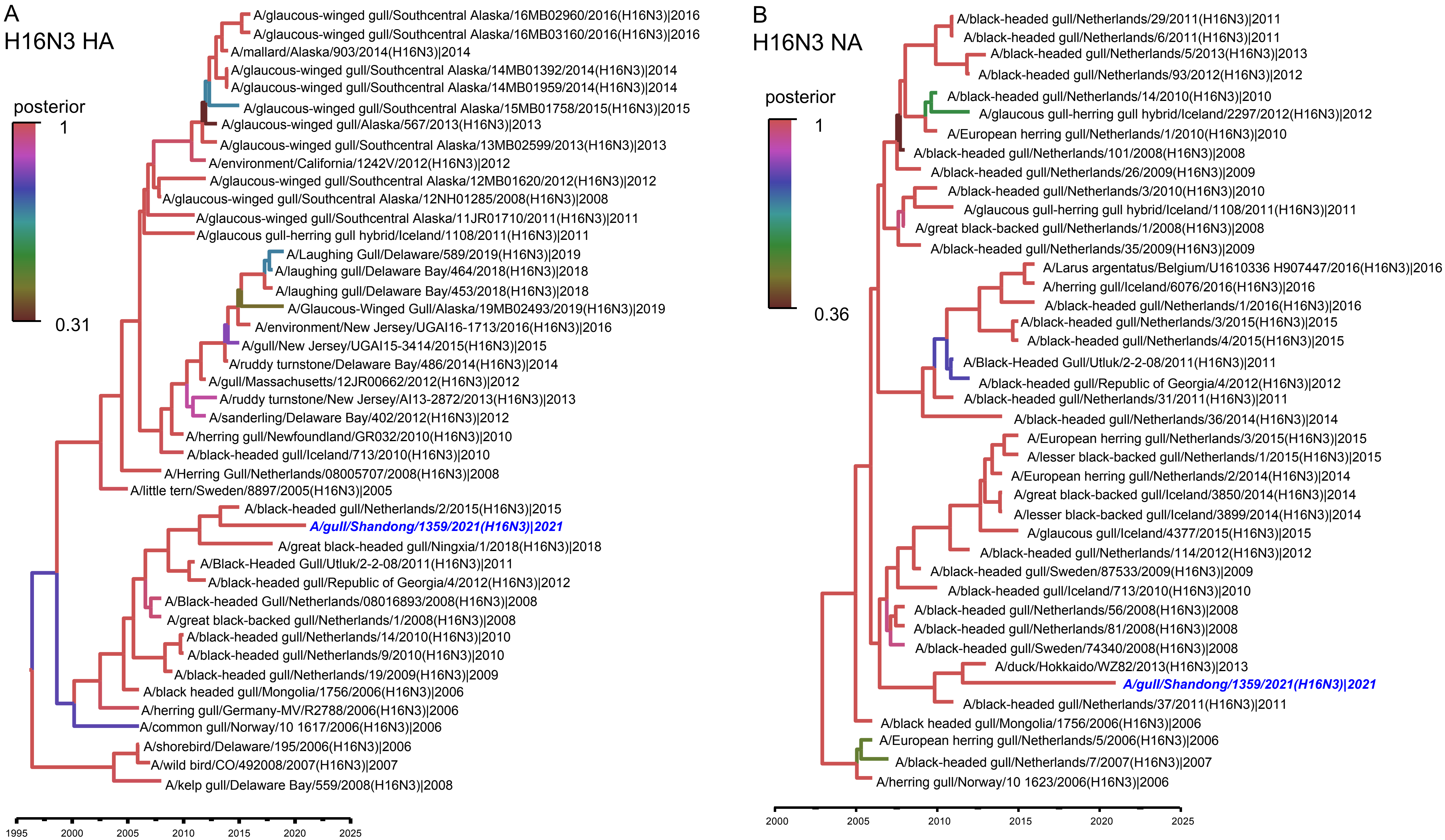
A
H16N3 HA
posterior
1
0.31
A/glaucous-winged gull/Southcentral Alaska/16MB02960/2016(H16N3)|2016
A/glaucous-winged gull/Southcentral Alaska/16MB03160/2016(H16N3)|2016
A/mallard/Alaska/903/2014(H16N3)|2014
A/glaucous-winged gull/Southcentral Alaska/14MB01392/2014(H16N3)|2014
A/glaucous-winged gull/Southcentral Alaska/14MB01959/2014(H16N3)|2014
A/glaucous-winged gull/Southcentral Alaska/15MB01758/2015(H16N3)|2015
A/glaucous-winged gull/Alaska/567/2013(H16N3)|2013
A/glaucous-winged gull/Southcentral Alaska/13MB02599/2013(H16N3)|2013
A/environment/California/1242V/2012(H16N3)|2012
A/glaucous-winged gull/Southcentral Alaska/12MB01620/2012(H16N3)|2012
A/glaucous-winged gull/Southcentral Alaska/12NH01285/2008(H16N3)|2008
A/glaucous-winged gull/Southcentral Alaska/11JR01710/2011(H16N3)|2011
A/glaucous gull-herring gull hybrid/Iceland/1108/2011(H16N3)|2011
A/Laughing Gull/Delaware/589/2019(H16N3)|2019
A/laughing gull/Delaware Bay/464/2018(H16N3)|2018
A/laughing gull/Delaware Bay/453/2018(H16N3)|2018
A/Glaucous-Winged Gull/Alaska/19MB02493/2019(H16N3)|2019
A/environment/New Jersey/UGAI16-1713/2016(H16N3)|2016
A/gull/New Jersey/UGAI15-3414/2015(H16N3)|2015
A/ruddy turnstone/Delaware Bay/486/2014(H16N3)|2014
A/gull/Massachusetts/12JR00662/2012(H16N3)|2012
A/ruddy turnstone/New Jersey/AI13-2872/2013(H16N3)|2013
A/sanderling/Delaware Bay/402/2012(H16N3)|2012
A/herring gull/Newfoundland/GR032/2010(H16N3)|2010
A/black-headed gull/Iceland/713/2010(H16N3)|2010
A/Herring Gull/Netherlands/08005707/2008(H16N3)|2008
A/little tern/Sweden/8897/2005(H16N3)|2005
A/black-headed gull/Netherlands/2/2015(H16N3)|2015
A/gull/Shandong/1359/2021(H16N3)|2021
A/great black-headed gull/Ningxia/1/2018(H16N3)|2018
A/Black-Headed Gull/Utluk/2-2-08/2011(H16N3)|2011
A/black-headed gull/Republic of Georgia/4/2012(H16N3)|2012
A/Black-headed Gull/Netherlands/08016893/2008(H16N3)|2008
A/great black-backed gull/Netherlands/1/2008(H16N3)|2008
A/black-headed gull/Netherlands/14/2010(H16N3)|2010
A/black-headed gull/Netherlands/9/2010(H16N3)|2010
A/black-headed gull/Netherlands/19/2009(H16N3)|2009
A/black headed gull/Mongolia/1756/2006(H16N3)|2006
A/herring gull/Germany-MV/R2788/2006(H16N3)|2006
A/common gull/Norway/10 1617/2006(H16N3)|2006
A/shorebird/Delaware/195/2006(H16N3)|2006
A/wild bird/CO/492008/2007(H16N3)|2007
A/kelp gull/Delaware Bay/559/2008(H16N3)|2008
1995
2000
2005
2010
2015
2020
2025
B
H16N3 NA
posterior
1
0.36
A/black-headed gull/Netherlands/29/2011(H16N3)|2011
A/black-headed gull/Netherlands/6/2011(H16N3)|2011
A/black-headed gull/Netherlands/5/2013(H16N3)|2013
A/black-headed gull/Netherlands/93/2012(H16N3)|2012
A/black-headed gull/Netherlands/14/2010(H16N3)|2010
A/glaucous gull-herring gull hybrid/Iceland/2297/2012(H16N3)|2012
A/European herring gull/Netherlands/1/2010(H16N3)|2010
A/black-headed gull/Netherlands/101/2008(H16N3)|2008
A/black-headed gull/Netherlands/26/2009(H16N3)|2009
A/black-headed gull/Netherlands/3/2010(H16N3)|2010
A/glaucous gull-herring gull hybrid/Iceland/1108/2011(H16N3)|2011
A/great black-backed gull/Netherlands/1/2008(H16N3)|2008
A/black-headed gull/Netherlands/35/2009(H16N3)|2009
A/Larus argentatus/Belgium/U1610336 H907447/2016(H16N3)|2016
A/herring gull/Iceland/6076/2016(H16N3)|2016
A/black-headed gull/Netherlands/1/2016(H16N3)|2016
A/black-headed gull/Netherlands/3/2015(H16N3)|2015
A/black-headed gull/Netherlands/4/2015(H16N3)|2015
A/Black-Headed Gull/Utluk/2-2-08/2011(H16N3)|2011
A/black-headed gull/Republic of Georgia/4/2012(H16N3)|2012
A/black-headed gull/Netherlands/31/2011(H16N3)|2011
A/black-headed gull/Netherlands/36/2014(H16N3)|2014
A/European herring gull/Netherlands/3/2015(H16N3)|2015
A/lesser black-backed gull/Netherlands/1/2015(H16N3)|2015
A/European herring gull/Netherlands/2/2014(H16N3)|2014
A/great black-backed gull/Iceland/3850/2014(H16N3)|2014
A/lesser black-backed gull/Iceland/3899/2014(H16N3)|2014
A/glaucous gull/Iceland/4377/2015(H16N3)|2015
A/black-headed gull/Netherlands/114/2012(H16N3)|2012
A/black-headed gull/Sweden/87533/2009(H16N3)|2009
A/black-headed gull/Iceland/713/2010(H16N3)|2010
A/black-headed gull/Netherlands/56/2008(H16N3)|2008
A/black-headed gull/Netherlands/81/2008(H16N3)|2008
A/black-headed gull/Sweden/74340/2008(H16N3)|2008
A/duck/Hokkaido/WZ82/2013(H16N3)|2013
A/gull/Shandong/1359/2021(H16N3)|2021
A/black-headed gull/Netherlands/37/2011(H16N3)|2011
A/black headed gull/Mongolia/1756/2006(H16N3)|2006
A/European herring gull/Netherlands/5/2006(H16N3)|2006
A/black-headed gull/Netherlands/7/2007(H16N3)|2007
A/herring gull/Norway/10 1623/2006(H16N3)|2006
2000
2005
2010
2015
2020
2025

**Figure S1. Maximum Clade Credibility (MCC) trees of the HA and NA genes of the H16N3 viruses.** Bayesian time-measured phylogenetic trees of HA (A) and NA (B) were constructed with the BEAST software package (v1.10.4) and then visualized by using FigTree (v1.4.4). Branches are colored according to posterior probability, and the node bars indicate the 95% highest posterior density of the node height. Virus isolated in this study are shown in blue.

Figure S2

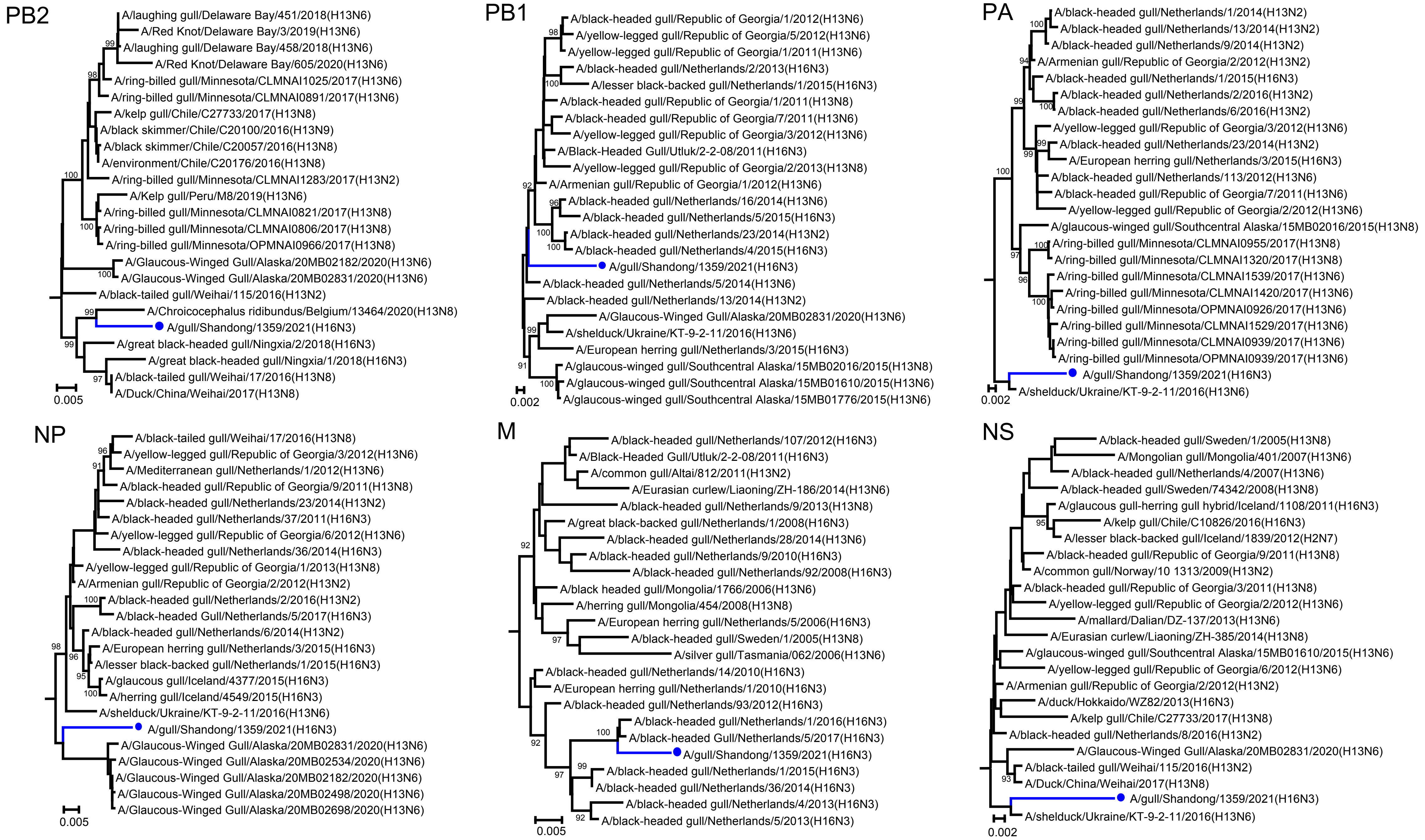
PB2
A/laughing gull/Delaware Bay/451/2018(H13N6)
A/Red Knot/Delaware Bay/3/2019(H13N6)
A/laughing gull/Delaware Bay/458/2018(H13N6)
A/Red Knot/Delaware Bay/605/2020(H13N6)
A/ring-billed gull/Minnesota/CLMNAI1025/2017(H13N6)
A/ring-billed gull/Minnesota/CLMNAI0891/2017(H13N6)
A/kelp gull/Chile/C27733/2017(H13N8)
A/black skimmer/Chile/C20100/2016(H13N9)
A/black skimmer/Chile/C20057/2016(H13N8)
A/environment/Chile/C20176/2016(H13N8)
A/ring-billed gull/Minnesota/CLMNAI1283/2017(H13N2)
A/Kelp gull/Peru/M8/2019(H13N6)
A/ring-billed gull/Minnesota/CLMNAI0821/2017(H13N8)
A/ring-billed gull/Minnesota/CLMNAI0806/2017(H13N8)
A/ring-billed gull/Minnesota/OPMNAI0966/2017(H13N8)
A/Glaucous-Winged Gull/Alaska/20MB02182/2020(H13N6)
A/Glaucous-Winged Gull/Alaska/20MB02831/2020(H13N6)
A/black-tailed gull/Weihai/115/2016(H13N2)
A/Chroicocephalus ridibundus/Belgium/13464/2020(H13N8)
A/gull/Shandong/1359/2021(H16N3)
A/great black-headed gull/Ningxia/2/2018(H16N3)
A/great black-headed gull/Ningxia/1/2018(H16N3)
A/black-tailed gull/Weihai/17/2016(H13N8)
A/Duck/China/Weihai/2017(H13N8)
0.005
PB1
A/black-headed gull/Republic of Georgia/1/2012(H13N6)
A/yellow-legged gull/Republic of Georgia/5/2012(H13N6)
A/yellow-legged gull/Republic of Georgia/1/2011(H13N6)
A/black-headed gull/Netherlands/2/2013(H16N3)
A/lesser black-backed gull/Netherlands/1/2015(H16N3)
A/black-headed gull/Republic of Georgia/1/2011(H13N8)
A/black-headed gull/Republic of Georgia/7/2011(H13N6)
A/yellow-legged gull/Republic of Georgia/3/2012(H13N6)
A/Black-Headed Gull/Utluk/2-2-08/2011(H16N3)
A/yellow-legged gull/Republic of Georgia/2/2013(H13N8)
A/Armenian gull/Republic of Georgia/1/2012(H13N6)
A/black-headed gull/Netherlands/16/2014(H13N6)
A/black-headed gull/Netherlands/5/2015(H16N3)
A/black-headed gull/Netherlands/23/2014(H13N2)
A/black-headed gull/Netherlands/4/2015(H16N3)
A/gull/Shandong/1359/2021(H16N3)
A/black-headed gull/Netherlands/5/2014(H13N6)
A/black-headed gull/Netherlands/13/2014(H13N2)
A/Glaucous-Winged Gull/Alaska/20MB02831/2020(H13N6)
A/shelduck/Ukraine/KT-9-2-11/2016(H13N6)
A/European herring gull/Netherlands/3/2015(H16N3)
A/glaucous-winged gull/Southcentral Alaska/15MB02016/2015(H13N8)
A/glaucous-winged gull/Southcentral Alaska/15MB01610/2015(H13N6)
A/glaucous-winged gull/Southcentral Alaska/15MB01776/2015(H13N6)
0.002
PA
A/black-headed gull/Netherlands/1/2014(H13N2)
A/black-headed gull/Netherlands/13/2014(H13N2)
A/black-headed gull/Netherlands/9/2014(H13N2)
A/Armenian gull/Republic of Georgia/2/2012(H13N2)
A/black-headed gull/Netherlands/1/2015(H16N3)
A/black-headed gull/Netherlands/2/2016(H13N2)
A/black-headed gull/Netherlands/6/2016(H13N2)
A/yellow-legged gull/Republic of Georgia/3/2012(H13N6)
A/black-headed gull/Netherlands/23/2014(H13N2)
A/European herring gull/Netherlands/3/2015(H16N3)
A/black-headed gull/Netherlands/113/2012(H13N6)
A/black-headed gull/Republic of Georgia/7/2011(H13N6)
A/yellow-legged gull/Republic of Georgia/2/2012(H13N6)
A/glaucous-winged gull/Southcentral Alaska/15MB02016/2015(H13N8)
A/ring-billed gull/Minnesota/CLMNAI0955/2017(H13N8)
A/ring-billed gull/Minnesota/CLMNAI1320/2017(H13N8)
A/ring-billed gull/Minnesota/CLMNAI1539/2017(H13N6)
A/ring-billed gull/Minnesota/CLMNAI1420/2017(H13N6)
A/ring-billed gull/Minnesota/OPMNAI0926/2017(H13N6)
A/ring-billed gull/Minnesota/CLMNAI1529/2017(H13N6)
A/ring-billed gull/Minnesota/CLMNAI0939/2017(H13N6)
A/ring-billed gull/Minnesota/OPMNAI0939/2017(H13N6)
A/gull/Shandong/1359/2021(H16N3)
A/shelduck/Ukraine/KT-9-2-11/2016(H13N6)
0.002
NP
A/black-tailed gull/Weihai/17/2016(H13N8)
A/yellow-legged gull/Republic of Georgia/3/2012(H13N6)
A/Mediterranean gull/Netherlands/1/2012(H13N6)
A/black-headed gull/Republic of Georgia/9/2011(H13N8)
A/black-headed gull/Netherlands/23/2014(H13N2)
A/black-headed gull/Netherlands/37/2011(H16N3)
A/yellow-legged gull/Republic of Georgia/6/2012(H13N6)
A/black-headed gull/Netherlands/36/2014(H16N3)
A/yellow-legged gull/Republic of Georgia/1/2013(H13N8)
A/Armenian gull/Republic of Georgia/2/2012(H13N2)
A/black-headed gull/Netherlands/2/2016(H13N2)
A/black-headed Gull/Netherlands/5/2017(H16N3)
A/black-headed gull/Netherlands/6/2014(H13N2)
A/European herring gull/Netherlands/3/2015(H16N3)
A/lesser black-backed gull/Netherlands/1/2015(H16N3)
A/glaucous gull/Iceland/4377/2015(H16N3)
A/herring gull/Iceland/4549/2015(H16N3)
A/shelduck/Ukraine/KT-9-2-11/2016(H13N6)
A/gull/Shandong/1359/2021(H16N3)
A/Glaucous-Winged Gull/Alaska/20MB02831/2020(H13N6)
A/Glaucous-Winged Gull/Alaska/20MB02534/2020(H13N6)
A/Glaucous-Winged Gull/Alaska/20MB02182/2020(H13N6)
A/Glaucous-Winged Gull/Alaska/20MB02498/2020(H13N6)
A/Glaucous-Winged Gull/Alaska/20MB02698/2020(H13N6)
0.005
M
A/black-headed gull/Netherlands/107/2012(H16N3)
A/Black-Headed Gull/Utluk/2-2-08/2011(H16N3)
A/common gull/Altai/812/2011(H13N2)
A/Eurasian curlew/Liaoning/ZH-186/2014(H13N6)
A/black-headed gull/Netherlands/9/2013(H13N8)
A/great black-backed gull/Netherlands/1/2008(H16N3)
A/black-headed gull/Netherlands/28/2014(H13N6)
A/black-headed gull/Netherlands/9/2010(H16N3)
A/black-headed gull/Netherlands/92/2008(H16N3)
A/black headed gull/Mongolia/1766/2006(H13N6)
A/herring gull/Mongolia/454/2008(H13N8)
A/European herring gull/Netherlands/5/2006(H16N3)
A/black-headed gull/Sweden/1/2005(H13N8)
A/silver gull/Tasmania/062/2006(H13N6)
A/black-headed gull/Netherlands/14/2010(H16N3)
A/European herring gull/Netherlands/1/2010(H16N3)
A/black-headed gull/Netherlands/93/2012(H16N3)
A/black-headed gull/Netherlands/1/2016(H16N3)
A/black-headed Gull/Netherlands/5/2017(H16N3)
A/gull/Shandong/1359/2021(H16N3)
A/black-headed gull/Netherlands/1/2015(H16N3)
A/black-headed gull/Netherlands/36/2014(H16N3)
A/black-headed gull/Netherlands/4/2013(H16N3)
A/black-headed gull/Netherlands/5/2013(H16N3)
0.005
NS
A/black-headed gull/Sweden/1/2005(H13N8)
A/Mongolian gull/Mongolia/401/2007(H13N6)
A/black-headed gull/Netherlands/4/2007(H13N6)
A/black-headed gull/Sweden/74342/2008(H13N8)
A/glaucous gull-herring gull hybrid/Iceland/1108/2011(H16N3)
A/kelp gull/Chile/C10826/2016(H16N3)
A/lesser black-backed gull/Iceland/1839/2012(H2N7)
A/black-headed gull/Republic of Georgia/9/2011(H13N8)
A/common gull/Norway/10 1313/2009(H13N2)
A/black-headed gull/Republic of Georgia/3/2011(H13N8)
A/yellow-legged gull/Republic of Georgia/2/2012(H13N6)
A/mallard/Dalian/DZ-137/2013(H13N6)
A/Eurasian curlew/Liaoning/ZH-385/2014(H13N8)
A/glaucous-winged gull/Southcentral Alaska/15MB01610/2015(H13N6)
A/yellow-legged gull/Republic of Georgia/6/2012(H13N6)
A/Armenian gull/Republic of Georgia/2/2012(H13N2)
A/duck/Hokkaido/WZ82/2013(H16N3)
A/kelp gull/Chile/C27733/2017(H13N8)
A/black-headed gull/Netherlands/8/2016(H13N2)
A/Glaucous-Winged Gull/Alaska/20MB02831/2020(H13N6)
A/black-tailed gull/Weihai/115/2016(H13N2)
A/Duck/China/Weihai/2017(H13N8)
A/gull/Shandong/1359/2021(H16N3)
A/shelduck/Ukraine/KT-9-2-11/2016(H13N6)
0.002

**Figure S2. Phylogenetic tree of the internal gene.** The phylogenetic trees of the six internal genes were constructed by MEGA 7.0 with Neighbor-Joining method. The sequence name with blue was the virus isolated in this study, and the sequences with black were download from the database.

Figure S3

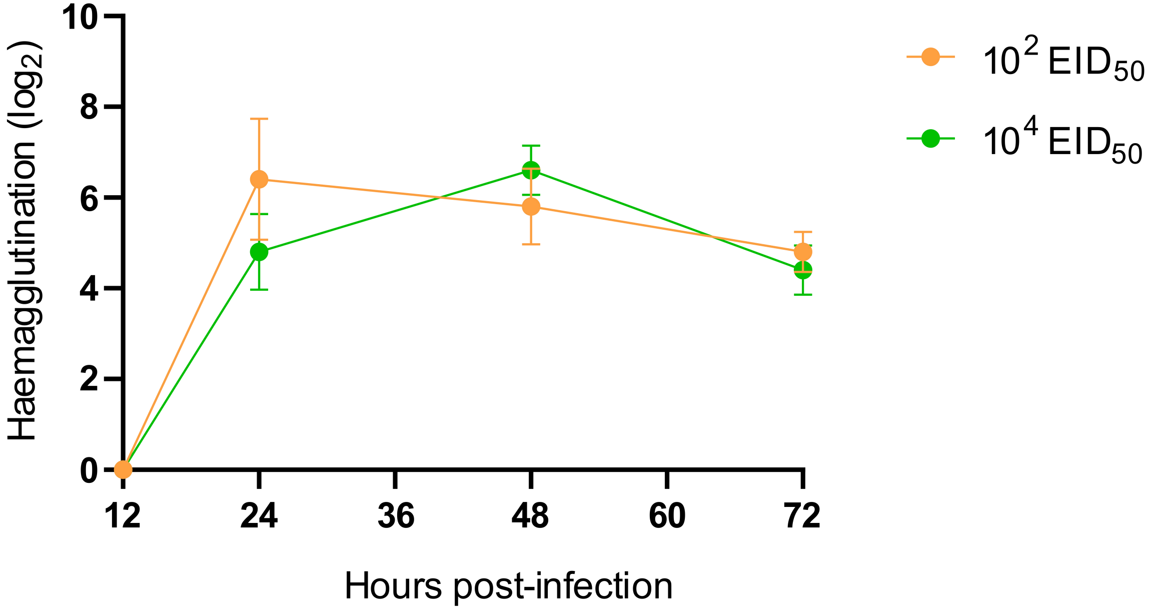


**Figure S3. Growth kenetics of the H16N3 virus in chicken embryonated eggs.** The viruses were inoculated in eggs in a volume with $10^2$ and $10^4$ $EID_{50}$, respectively. The haemagglutinin titer was detected by using the 0.5% chicken red blood cells. The data shown are the means of three replicates.

**Materials and methods**

**Ethics statement and facility**

The animal studies were carried out in strict accordance with the recommendations in the Guide for the Care and Use of Laboratory Animals of the Ministry of Science and Technology of the People's Republic of China. The protocols for chicken, duck, and mouse studies were approved by the Committee on the Ethics of Animal Experiments of Liaocheng University. All experiments with H16N3 virus were conducted in an animal biosafety level 2 (ABSL-2) facility. The animals used in this study were placed in a biological safety isolator. The researchers who worked with the animals wore N95 masks and disposable overalls.

**Experimental animals**

Six-week-old SPF female BALB/c mice were purchased from Jinan Pengyue Experimental Animal Breeding Co., Ltd. (Shandong, China). Three-week-old SPF chickens and ducks were purchased from Shandong Healthtech Laboratory Animal Breeding Co., Ltd.

**Sequences data analysis**

We downloaded all the HA and NA sequences of H16 viruses from Genbank and GISAID. The virus information, including the host, isolated site, isolation time and subtype were statistically analyzed and presented in the figure.

**Sample collection, virus identification and isolation**

Fresh fecal droppings of migratory gulls were collected and then placed into 2 ml of minimal essential medium supplemented with penicillin and streptomycin. Positive fecal samples were identified by PCR with specific M and HA (H5, H7) primers. The suspected H5- or H7-positive samples were transferred to an enhanced ABSL-3 facility in the Harbin Veterinary Research Institute of Chinese Academy of Agricultural Sciences for further virus identification and isolation, while the remaining suspected positive samples were injected into 10-day-old embryonated chicken eggs to isolate the viruses in an ABSL-2 laboratory at Liaocheng University. The HA subtype was determined by using the HA inhibition (HI) test and genetic sequencing, while the neuraminidase (NA) subtype was determined by PCR and genetic sequencing. The isolated viruses were stored in a -80 ℃ freezer.

**Genetic and molecular analysis**

The RNA of the viruses was extracted from the allantoic fluid of virus-infected eggs, and reverse transcription PCR was performed using gene-specific primers. The PCR products of each gene segment of H3N8 viruses were sequenced using specific sequencing primers

(primer sequences available upon request) at Sangon Biotech (Shanghai) Co., Ltd. The sequence data were compiled with the SEQMAN program (DNASTAR, Madison, WI) according to the reference sequences. The molecular markers at each segment were identified with the MegAlign program (DNASTAR, Madison, WI).

**Phylogenetic analysis**

Phylogenetic analysis was performed by employing the maximum likelihood method using the MEGA 7.0 ClustalW software package. The phylogenetic tree was constructed with the PHYLIP program of MEGA 7.0 software using the neighbor-joining algorithm. A bootstrap value of 1000 was used.

**Bayesian phylogenetic analysis**

Markov chain Monte Carlo (MCMC) trees with molecular clocks were constructed using BEAST (V2.6.2) software to study the evolutionary history of H3N8 viruses in wild birds of eastern China. Bayesian systematic analyses of the HA and NA genes were constructed using the general time reversible (GTR) model and relaxed clock log normal and 100-million step MCMC methods. Tracer (v1.7.1) was used to observe whether the parameters converged. MCMC tree files were obtained using TreeAnnotator software, with 10% burn-in. Figtree (v1.4.4) was used to generate the MCMC trees with a time scale.

**Animal experiments**

**Mice** Six-week-old female SPF mice (eight animals in each group) were inoculated with $10^6$ $EID_{50}$ of the virus in a volume of 50 µl. Three mice were euthanized on day 3 pi, and nasal turbinate, lung, spleen, kidney, and brain tissues were collected for viral titration in eggs. The remaining five mice were monitored daily for 14 days for weight loss and survival. Mice inoculated with PBS were established as a control group and used to observe body weight changes.

**Chickens** Three chickens from each group were inoculated with $10^6$ $EID_{50}$ of the virus in a volume of 200 µl. Brain, tracheal, lung, liver, spleen, pancreatic, kidney, intestinal, rectal, and bursa of Fabricius tissues of the chickens were collected for viral titration in eggs at day 3 pi. For the transmission study, five chickens from each group were inoculated with $10^6$ $EID_{50}$ of the virus in a volume of 200 µl. Another five naive chickens were placed into the same isolator at 24 hpi. The oropharyngeal and cloacal swabs of the chickens were collected on days 1, 3, 5, 7, 9, and 11 pi, respectively. The viral titers of the swabs were titrated in eggs.

Chicken serum was collected on days 10, 15, and 21 pi, and the antibody titer was determined by the HI test. The chickens were then euthanized on day 21 pi.

**Ducks** Three ducks from each group were inoculated with $10^6$ $EID_{50}$ of the virus in a volume of 200 μl. Brain, tracheal, lung, liver, spleen, pancreatic, kidneys, intestinal, rectal, and bursa of Fabricius tissues of the ducks were collected for viral titration in chicken eggs at day 3 pi. For the transmission study, three ducks from each group were inoculated with $10^6$ $EID_{50}$ of the virus in a volume of 200 μl. Another five naive ducks were placed into the same isolator at 24 hpi. The oropharyngeal and cloacal swabs of the ducks were collected on days 1, 3, 5, 7, 9, and 11 pi, respectively. The viral titers of the swabs were titrated in eggs. Duck serum was collected on days 10, 15, and 21 pi, and the antibody titer was determined by the HI test. The ducks were then euthanized on day 21 pi.

**Statistical Analysis**

The analysis of growth kinetics and virus replication of the H16N3 virus were performed with GraphPad Prism 8. Statistical significance of comparisons between two groups was determined with the Student's t-test. $p$ less than 0.05 were considered statistically significant.
